# Comparative Analysis of Body Image Dissatisfaction, Depression, and Health-Related Quality of Life in Adults with Type 1 Diabetes: A Case-Control Study

**DOI:** 10.3390/nu15183938

**Published:** 2023-09-11

**Authors:** Stephen J. Inns, Amanda Chen, Helen Myint, Priyanka Lilic, Crispin Ovenden, Heidi Y. Su, Rosemary M. Hall

**Affiliations:** 1 Te Whatu Ora Health New Zealand, P.O. Box 793, Wellington 6140, New Zealandcrispin.ovenden@cdhb.health.nz (C.O.);; 2 Department of Medicine, University of Otago, P.O. Box 7343, Wellington 6242, New Zealand

**Keywords:** type 1 diabetes, case-control, age and gender matched, body image dissatisfaction, quality of life, depression and anxiety

## Abstract

(1) Objective: This case-control study investigated body image dissatisfaction, depression, and health-related quality of life (HRQoL) in adults with type 1 diabetes. (2) Methods: A total of 35 adults with diabetes and an equal number of age- and gender-matched controls were included. Assessment tools used were the Body Image Disturbance Questionnaire (BIDQ), the Hospital Anxiety and Depression Scale (HADS), and the RAND 36-Item Health Survey. Both quantitative and qualitative data were analyzed. (3) Results: Body image dissatisfaction did not differ significantly between the groups. However, adults with diabetes reported higher levels of depression (*p* = 0.002) and lower scores for physical health (*p* = 0.015) and general health (*p* < 0.001) on the HRQoL measure. Qualitative analysis identified common themes related to physical disturbance, effect on activities, and psychosocial concerns. (4) Conclusions: Despite similar body image dissatisfaction, adults with type 1 diabetes exhibited increased depression and reduced HRQoL. These findings emphasize the need to integrate psychological well-being into type 1 diabetes management. They also support further research into the impact of body image dissatisfaction in T1D and potential interventions to address it.

## 1. Introduction

Type 1 diabetes mellitus (T1D) is a chronic autoimmune disease characterized by the destruction of pancreatic beta-cells, resulting in absolute insulin deficiency [1,2]. Managing T1D is complex and requires lifelong treatment, including careful dietary management, blood glucose monitoring, and insulin replacement [3,4].

The global incidence of T1D has been rising, with New Zealand also witnessing an increasing trend [5]. This rise not only poses challenges in medical management but also intensifies the societal implications of chronic illnesses. Physiologically, individuals with T1D undergo a myriad of changes, including potential weight fluctuations due to insulin therapy, which can profoundly affect one’s perception of their body [6].

In the global context, where societal values and cultural norms increasingly emphasize body image and physical appearance due to influences from media, fashion, and popular culture [7], T1D patients may grapple more with societal expectations. This worldwide cultural environment might intensify the body image dissatisfaction experienced by T1D patients, underscoring the urgency to address this issue universally and understand its nuances across different regions and cultures.

Addressing body image dissatisfaction is not just about improving one’s perception of their body. It is crucial from a mental health perspective. Untreated body image issues can lead to severe mental health challenges, including depression, anxiety, and even the onset of eating disorders, further complicating the management of T1D [8].

Numerous chronic diseases are recognized for their substantial influence on body image dissatisfaction (BID). Inflammatory bowel disease (IBD), for instance, has been associated with weight changes and altered self-perception due to the visibility of surgical outcomes [9,10]. Cancer treatments, especially those for breast or testicular cancers, often lead to notable physical alterations like scarring or chemotherapy-induced alopecia, with associated psychological impacts [11]. The pervasive influence of such diseases on BID accentuates the significance of our investigation into its nuances within the T1D community.

In 1971, Kaufman and Hersher introduced the idea that the combined effects of the disease could lead to alterations in body image perception among young people with T1D [12]. A study by Steel et al. examined body image perception at the time of T1D diagnosis and twelve months later. The findings revealed significant increases in body dissatisfaction scores among female subjects, while similar changes were not observed to the same extent in males, despite experiencing similar weight gain [13].

Erkolahti et al. conducted a study comparing body image perception between a group of 23 adolescents with T1D and 26 adolescents in a control group [14]. The researchers found that the adolescents with T1D had poorer body image ratings based on the “Offer Self-Image Questionnaire”. However, these differences were not statistically significant, and no significant differences were observed between boys and girls.

In contrast, a study in 2008 involving 143 adolescents with T1D and 4746 adolescent controls found that both male and female adolescents with T1D were significantly more satisfied with their weight compared to the control group [15]. Interestingly, no significant differences were found in the use of extreme unhealthy weight control behaviors (e.g., vomiting, taking diet pills, laxatives, diuretics, or manipulating insulin) between the two groups. However, it is concerning that a portion of the adolescents with T1D reported skipping insulin (10.3% of girls and 1.4% of boys) or taking less insulin than recommended (7.4% of girls and 1.4% of boys) in the past year as a means of weight control.

These studies did not demonstrate that young patients with T1D might have more problems with body image dissatisfaction than their peers. However, they did raise the concern that, in some individuals with T1D, body image dissatisfaction might lead to disordered behavior, in particular the use of insulin avoidance as a weight control strategy.

Other case-control studies have consistently reported low body image satisfaction in children and adolescents with T1D, but have not found significant differences in body image dissatisfaction between T1D patients and controls [6,16,17,18,19]. However, these studies have identified a subset of individuals with T1D who exhibit concerning behaviors, such as insulin omission, as a strategy to manipulate their weight [6,15,19,20,21]. It is important to note that body dissatisfaction has been identified as a significant predictor of eating disorders [22].

Given these findings, it is crucial to address the potential risks of eating disorders, particularly the dangerous behavior of insulin omission, in individuals with T1D. To better understand this risk and its prevalence in the local population, it is essential to investigate the incidence of body image dissatisfaction among individuals with T1D in New Zealand. While previous studies have shed light on the relationship between body image dissatisfaction and T1D, no study has specifically examined this issue in New Zealand patients with T1D. Lunt, a New Zealand diabetologist, highlighted the risk of insulin omission as a weight control strategy in women with T1D [23]. Moreover, a study conducted in Christchurch, New Zealand, assessed a group of women with T1D, revealing a strong relationship between aspects of eating disturbance and psychological control, which significantly impacted metabolic control [24].

Therefore, the primary objective of this study was to investigate the prevalence of body image dissatisfaction among individuals with T1D from Lower Hutt, New Zealand, compared to age- and gender-matched healthy controls. A validated body image dissatisfaction inventory was utilized to assess and compare body image perceptions in both groups, alongside validated measures of physical and psychological wellbeing. By addressing this research gap, we aimed to contribute to a better understanding of the relationship between body image dissatisfaction and T1D, and ultimately inform interventions and support strategies to improve the well-being of individuals living with T1D.

## 2. Materials and Methods

This cross-sectional, prospective study recruited consecutive patients with Type 1 diabetes (T1D) who attended the diabetes clinic at Hutt Valley Hospital, New Zealand. To be eligible for participation, patients had to meet the following inclusion criteria: be over the age of 16 years and have a confirmed diagnosis of T1D for more than six months. We collected data from participants at a single time point, and we did not follow them longitudinally.

Matching of Subjects: In this study, we matched the participants with T1D and the healthy controls based on age and gender alone. Age and gender have been identified as important factors that can influence body image dissatisfaction (BID) across different populations. Previous research has indicated that age is inversely related to body dissatisfaction, suggesting that younger individuals may experience higher levels of body dissatisfaction than older individuals [25]. Moreover, gender differences in BID have been widely documented in the literature, with females generally reporting higher levels of BID compared to males [26]. Additionally, studies on children have shown that parent’s attitudes towards body size can impact their child’s body image [27]. Given this evidence, it was deemed appropriate to match subjects based on these two variables.

Age- and gender-matched healthy controls were recruited at the same time and were made up of people who volunteered in response to notices placed in hospital newsletters at the same center.

People with T1D were excluded from the study if they had a co-existing chronic gastrointestinal disease or were pregnant. Healthy controls were only included if they had no chronic disease diagnosis; they were excluded if they were pregnant.

Written informed consent was obtained prior to entering the study. This study was conducted in compliance with relevant NZ legislation and the Health Information Privacy Code 1994. Ethical approval was gained from the University of Otago Human Ethics Committee (reference number: H16/108).

Demographic data were collected for all participants, including age; gender; ethnicity (using NZ census categories); co-morbidities; medication history; smoking history; relationship status; and body mass index. For the participants with T1D, data pertaining to their diabetes were collected.

**Figure 1:**
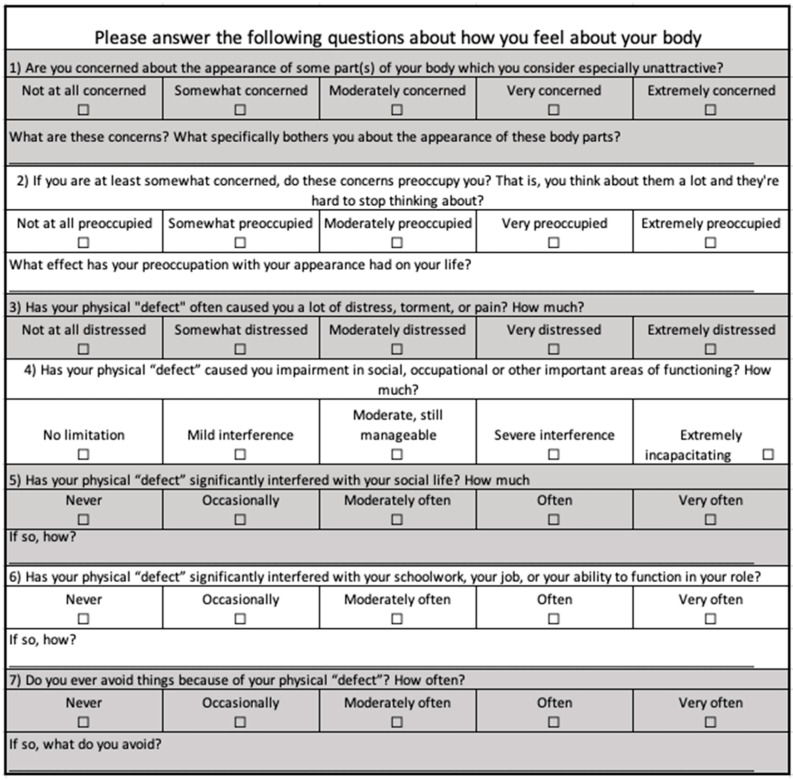
The Body Image Disturbance Questionnaire.

All participants completed three questionnaires. For insight into body image dissatisfaction, we used the Body Image Disturbance Questionnaire (BIDQ), a validated seven-item questionnaire that allows an individual to rank their appraisal of aspects of their body image on a continuous scale [28]. The result is reported as the mean score from the 7 individual questions, the BIDQ score (Figure 1).

The participants completed the Hospital Anxiety and Depression Score (HADS), a 14-question psychological screening tool, which has been validated for comparisons across clinical groups, as well as being well correlated with disease course, responses to treatment, and quality of life measures [29].

Study participants also completed the RAND 36 health related quality of life measure, an extensively used and well validated tool designed to capture quality of life by examining a range of domains including physical functioning; role limitations due to physical ill health; bodily pain; social functioning; general mental health (psychological distress and well-being); role limitations due to emotional problems; energy levels; and general health perceptions [29].

Participants were issued a unique study number. All completed questionnaires were labelled with the study number only, without any other identifying information.

### 2.1. Statistical Methods

The data were analyzed using SPSS Statistics for Windows, Version 28.0 (IBM SPSS Statistics for Windows, Version 23.0. Armonk, NY, USA: IBM Corp). The primary analysis compared the BIDQ score between the two groups using the Student’s *t*-test. Other continuous outcomes were also compared using the Student’s *t*-test. Smoking status was compared using Fisher’s Exact Test, and other categorical variables with more than 2 levels were compared using the Fisher–Freeman–Halton exact test. The BIDQ mean score in healthy individuals was previously shown to be 1.57 (s.d. 0.6, *n* = 53) for men and 1.81 (s.d. 0.67, *n* = 198) for women [30]. We determined the sample size calculation based on several factors. Considering an estimated distribution of 50% males in our sample, we calculated the combined mean to be 1.69 with a standard deviation of 0.65. We aimed to detect a clinically significant difference of 0.5 with varying levels of statistical power. Initial power calculations suggested that 27 participants in each group would provide 80% power, and 36 participants would offer 90% power. Our final sample size of 35 per group approached but did not reach the higher power target. This should be considered when interpreting the results.

### 2.2. Qualitative Methods

In addition to the 7 quantitative questions, the inclusion of qualitative data in the form of 5 open-ended questions in the Body Image Disturbance Questionnaire was crucial for capturing a comprehensive understanding of the factors influencing body image satisfaction in individuals with chronic disease. This approach aligns with the principles of grounded theory methodology, which has been widely recognized for its ability to explore complex phenomena in-depth [31].

To ensure a rigorous analysis of the qualitative data, we adopted a grounded theory approach consistent with established methodologies [32]. The analysis process involved multiple iterative steps to ensure reliability and comprehensiveness. Two experienced physicians independently evaluated the participant responses, employing triangulation methodology to enhance the validity of the findings [33]. Through iterative discussions and consensus-building, major physical, psychological, and social themes, along with their respective subgroups, were identified.

The use of grounded theory methodology in analyzing qualitative data from the Body Image Disturbance Questionnaire has been successfully employed in previous research, particularly in patients with inflammatory bowel disease [34]. This methodological choice allowed for a systematic exploration of the data, capturing the nuances and complexities inherent in the participants’ body image perceptions.

## 3. Results

### 3.1. Quantitative Analysis

A total of 70 subjects were recruited, 35 patients who attended the diabetes clinic at Hutt Valley Hospital, New Zealand, and 35 age- and gender-matched controls. No difference between groups in smoking status, relationship status, or BMI was found (Table 1). There were differences in ethnicity between groups, with a larger proportion of people identifying as Asian in the control group.

**Figure 2:**
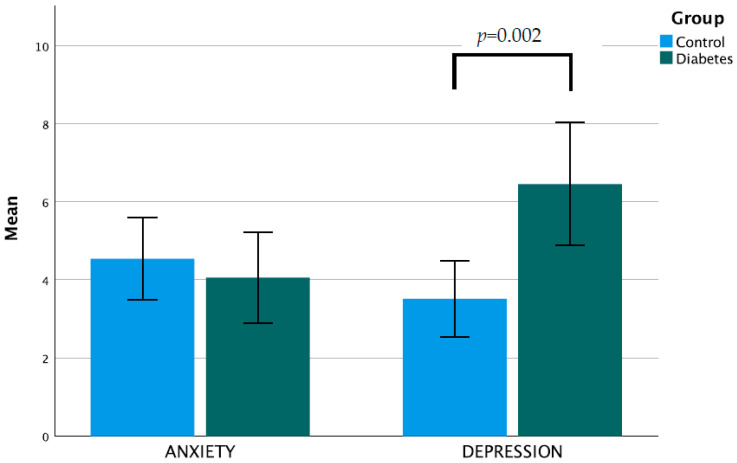
Mean Hospital Anxiety and Depression Score compared between groups (error bars show 95% confidence intervals).

**Figure 3:**
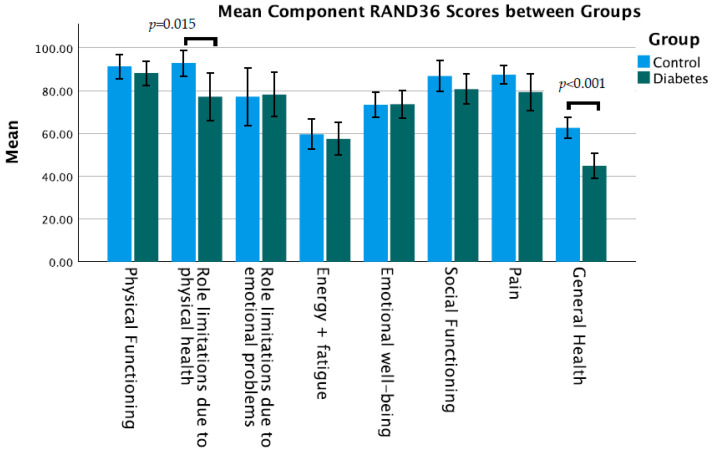
Mean RAND36 quality of life component scores compared between groups (error bars show 95% confidence intervals).

The main outcome measure, the mean BIDQ score, was not significantly different between people with T1D and controls (1.74 vs. 1.49, *p* = 0.096) (Table 2). Other measures of well-being, however, did differ between groups, particularly the depression component of the HADS (6.45 vs. 3.51, *p* = 0.002) (Figure 2), and the “role limitations due to physical health” (77.1 vs. 92.9, *p* = 0.015) and “general health” (44.7 vs. 62.6, *p* < 0.001) components of the RAND 36 (Figure 3). The anxiety component of the HADS and other components of the RAND 36 were not significantly different for people with T1D (Table 2).

### 3.2. Qualitative Analysis

All 70 subjects completed questions 1 to 7 of the BIDQ. Of the subjects with T1D, 24 (69%) made qualitative statements regarding body image in response to the five open ended questions. Of the control subjects, 23 (66%) made qualitative statements. As seen in Table 3, the themes that arose were closely shared by people with T1D and controls. Concern regarding weight was evident, particularly weight gain. Only in the T1D group were three subjects concerned at low weight. As could be expected, the effect of insulin injection and changes at injection sites was a concern for people with T1D but not controls. In both groups, concerns about appearance affected clothing choice and exercise, with a particular concern being swimwear and going to the beach or pool. In both groups, a lack of focus on tasks related to physical concerns was expressed, but, as would be expected, people with T1D expressed the additional concern of how hypoglycemia affects their performance. Selected qualitative responses are summarized in Table 4.

## 4. Discussion

This cross sectional, prospective, case-control study aimed to explore body image dissatisfaction and its association with psychological well-being in New Zealanders with Type 1 Diabetes (T1D) compared to healthy controls. Our findings show that individuals with T1D experience similar levels of body image dissatisfaction compared to the control group. Qualitative analysis further reveals that the physical and psychosocial effects of body image dissatisfaction in people with T1D are comparable to those experienced by the controls. However, a small subset of individuals with T1D expressed concerns related to low weight, concerns about insulin injection sites, and the impact of disease on performance, which were not expressed in the control group.

In our qualitative findings, concerns arising from insulin injection and changes at injection sites were distinctly expressed by participants with T1D. This likely references the phenomenon of lipohypertrophy, a complication from repeated insulin injections. The visual and tactile changes that come with lipohypertrophy can aggravate body image concerns in an individual, particularly when such manifestations are not commonly experienced by the general populace. Similarly, the uniquely T1D concern of how hypoglycemia affects performance points to a broader psychological burden. The anxiety stemming from potentially visible and disruptive hypoglycemic episodes can amplify body image concerns and induce behavioral changes, like social withdrawal.

While our sample had a greater number of females than males, the difference was not large (15 males vs. 20 females in each group). Despite the overarching similarity in the BIDQ scores between the T1D and control groups, our qualitative analysis subtly highlighted gendered concerns, like clothing choices and apprehension about swimwear or pool visits, which inherently tie into societal body image pressures. This broader societal narrative around body image may be intensified for women with T1D. The qualitative feedback underscores the need for interventions to be gender-sensitive, accounting for the unique experiences of females within the T1D cohort.

Despite the similar rates of body image dissatisfaction between the two groups, individuals with T1D reported significantly higher levels of depression and lower scores for “role limitations due to physical health” and “general health” in the RAND 36 quality of life score. These findings are concerning, as exercise avoidance was reported among individuals with T1D, despite the well-known benefits of exercise for this population [35]. Lower general health scores align with the reduced life expectancy observed in people with T1D, with an average of 12.2 years of life lost compared to the general population [36]. While the study did not specifically address longevity concerns, behaviors associated with depression and body image dissatisfaction may contribute to deteriorations in glucose management and the development of complications.

Previous studies have consistently demonstrated the association between body image dissatisfaction and psychological well-being, including depression and anxiety, across various disease populations [37]. Meta-analyses of this literature indicate that body appreciation is inversely associated with general psychopathology [38]. Similar associations have been observed in breast cancer [39], post-partum depression [40], systemic lupus erythematosus [41], and inflammatory bowel disease [10]. Notably, body dissatisfaction has also been linked to levels of inflammatory biomarkers, independent of weight loss, in patients undergoing a weight management program [42]. Such an association may hold significance in the context of a long-term illness like T1D.

This study, although conducted with a relatively small sample size, has the advantage of comparing patients with T1D to age- and gender-matched controls, as opposed to relying on normative values. This approach allowed for a more meaningful comparison and facilitated comparative qualitative analysis of the physical and psychological factors contributing to body image dissatisfaction. It is important to note, however, that while participants were matched in terms of age and gender, there was a higher proportion of Asian individuals in the control group compared to the T1D group. Despite these differences in ethnicity, the BMI remained comparable between the groups.

The potential influence of ethnicity on body image dissatisfaction and BMI should be considered when interpreting the results of our study. Cultural and societal norms have been shown to impact body image perceptions, and these norms may vary across ethnic groups [43,44,45]. For example, Olson et al. (2015) reported that individuals of different ethnicities had similar levels of body shape concerns, except for White individuals, who had higher body shape concerns compared to Asian/Pacific Islanders [44]. Mikolajczyk et al. (2012) observed ethnic differences in body satisfaction among U.S. schoolchildren and noted that African-American students had more positive body perceptions compared to other ethnic groups, but this positive perception decreased substantially in African-American boys during adolescence [45]. Additionally, Fitzgibbon et al. (2012) reported that White women experienced body dissatisfaction at a lower BMI level compared to Black and Hispanic women [43]. The ethnicity mismatch between the T1D and control groups in our study might affect the generalizability of our results. Further research is needed to explore the interplay between ethnicity, body image dissatisfaction, and BMI in larger, ethnically diverse populations, particularly among individuals with T1D.

Considering the implications of our findings, it is evident that our participants with T1D have different experiences of physical and psychological wellbeing than those in our control group. While we did not demonstrate a statistically significant difference in BIDQ between participants with T1D and controls, there was a trend towards increased BIDQ with T1D, which was more pronounced in females than males. Psychological interventions have shown promise in improving body dissatisfaction [46], while interventions specifically focused on body image dissatisfaction have been effective in reducing depression in adolescents [47].

Our study underscores the multifaceted nature of body image dissatisfaction in individuals with T1D. With concerns ranging from weight (both gain and the unique T1D concern of low weight) to insulin injection site changes, there’s a clear need for interventions to encompass a broad spectrum of concerns. Interventions that integrate practical T1D management strategies, such as proper insulin injection technique to mitigate lipohypertrophy, with cognitive–behavioral components can offer comprehensive support. Furthermore, given that concerns around clothing choice and beach or pool visits were shared across both T1D and control groups, body image interventions might also benefit from segments focusing on fostering body positivity in communal settings.

Future studies further examining the association between body image dissatisfaction and the occurrence of lower physical and psychological well-being in larger samples of patients with T1D are warranted. Such studies could incorporate treatment paradigms that involve interventions aimed at improving body image satisfaction, to determine whether this would benefit patients with T1D. Such studies would allow conclusions regarding the value of incorporating such measures into routine clinical care and highlight the importance of psychological support as a part of the management of T1D.

In conclusion, our study sheds light on the prevalence of body image dissatisfaction in New Zealanders with T1D and highlights the effect of T1D on psychological and physical well-being. Despite comparable levels of body image dissatisfaction between individuals with T1D and the control group, T1D participants reported higher levels of depression and lower scores on quality-of-life measures. These findings emphasize the importance of addressing psychological factors in the management of T1D.

## Figures and Tables

**Table 1:** Participant demographics.

	Diabetes	Control	
Mean Age years (s.d.)	37.1 (13.7)	37.7 (13)	*p* = 0.84
Gender (F:M)	20:15	20:15	
Mean BMI kg/m^2^ (s.d.)	27.4 (4.5) *	25.3 (3.8)	*p* = 0.05
**Smoking Status**			*p* = 0.11
Nonsmoker	35	31	
Current smoker	0	4	
**Relationship status**			*p* = 0.57
De facto/Married	24	21	
Single	9	13	
Unknown	2	1	
**Ethnicity**			*p* < 001
NZ European	32	12	
Māori	1	3	
Pacific Peoples	1	1	
Asian	1	17	
Other Ethnicity	0	2	

* The weight of one participant in the T1D group was not available. Categorical variables in bold with subcategories below.

**Table 2:** Detailed results of the BIDQ, RAND, and HADS analyses.

	Diabetes	Control	*p* Value
BIDQ overall	1.74	1.49	0.1
BIDQ males	1.36	1.28	0.29
BIDQ females	2.02	1.65	0.08
Physical functioning	88.1	91.3	0.43
Role limitations due to physical health	77.1	92.9	0.015
Role limitations due to emotional problems	78.1	77.1	0.91
Energy + fatigue	57.4	59.6	0.68
Emotional well-being	73.6	73.4	0.96
Social functioning	80.7	86.8	0.23
Pain	79.3	87.4	0.09
General health	44.7	62.6	<0.001
HADS anxiety	4.06	4.54	0.53
HADS depression	6.45	3.51	0.002

**Table 3:** Comparison of reports of qualitative themes between groups.

Code	Response Category	Diabetes Group No. Responses	Control Group No. Responses
Physical disturbance			
1a.	Concern about excess weight	11	7
1b.	Concern about adipose tissue in one area	11	14
1c.	Concern about low weight	3	0
1d.	Effect on clothing	15	15
1e.	Other physical concern	10	14
2. Effect on activities			
2a.	Concern regarding swimming pool and beach	9	15
2b.	Concern regarding exercising	6	3
3. Psychosocial concerns			
3a.	Fear/anxiety of public places generally	9	7
3b.	Concern about being different	3	1
3c.	Effect on self-esteem/confidence	1	8
3d.	Effect on mood	3	0
3e.	Concern regarding what others think	1	1
3f.	Effect on interpersonal relationships	0	1
3g.	Effect on performance/work/study	5	2

**Table 4:** Selected qualitative responses.

Diabetes Group	Control Group
Are you concerned about the appearance of some part(s) of your body, which you consider especially unattractive? What are these concerns? What specifically bothers you about the appearance of these body parts?	
“Recently [I] have gained weight which bothers me” (20, F, NZ European; code 1a) “I dislike how skinny my abdomen is” (18, F, Samoan; code 1c) “Weight around where I inject insulin” (31, F, NZ European; code 1a, 1b)	“[My] stomach and thighs and buttocks appear to have increased in size in the last 5 years” (57, F, NZ European; code 1b) “Scarring on the face/uneven skin tone” (23, F, Indian; code 1e)
What effect has your preoccupation with your appearance had on your life?	
“Starting to think more about food and exercise” (20, F, NZ European, code 2b) “I get quite anxious with what I wear” (18, F, Samoan, code 1d) “Sometimes feel sad/upset that I’m not small” (24, F, NZ European; code 3d)	“Change in type of clothing—not so many close fitting” (57, F, NZ European; code 1d) “Feel self-conscious” (54, F, Māori; code 3c)
Has your physical “defect” significantly interfered with your social life? If so, how?	
“Especially summer in swimsuits and less clothing” (35, F, NZ European; code 2a, 1d) “Affecting general confidence in new situations” (31, M, NZ European; code 3a)	“Not comfortable swimming” (57, F, NZ European; code 2a) “Too embarrassed to attend gatherings” (51, F, NZ European; code 3a)
Has your physical “defect” significantly interfered with your schoolwork, your job, or your ability to function in your role? If so, how?	
“Low blood sugars, need to stop what I’m doing” (57, F, NZ European; code 3g) “Felt down and unable to concentrate” (22, F, NZ European; code 3g)	“Not focusing on course work” (21, F, Other Ethnicity; code 3g)
Do you ever avoid things because of your physical “defect”? If so, what do you avoid?	
“Going to the beach/pools” (35, F, NZ European; code 2a) “Avoid exercising in public if at all” (22, F, NZ European; code 2b) “Certain clothes I would like to wear” (57, F, NZ European; code 1d)	“Swimming mainly” (57, F, NZ European; code 2a) “[I] give up on exercise sometimes” (30, F, Asian; code 2b) “Wearing dresses and shorts” (24, F, Samoan; code 1d)

## Data Availability

The data presented in this study are available on request from the corresponding author. The data are not publicly available due to privacy and ethical restrictions.
